# Editorial: Executive functions in psychiatric science, clinical practice and beyond: A Transdiagnostic Window on Functional Heterogeneity

**DOI:** 10.3389/fpsyt.2025.1743127

**Published:** 2025-12-09

**Authors:** Predrag Petrovic, Charlotte Borg Skoglund, Edmund Sonuga-Barke

**Affiliations:** 1 Department of Clinical Neuroscience, Karolinska Institutet, Center for Cognitive and Computational Neuropsychiatry (CCNP), Stockholm, Sweden; 2 Department of Clinical Neuroscience, Karolinska Institutet, Center for Psychiatry Research (CPF), Stockholm, Sweden; 3 Department of Women’s and Children’s Health, Uppsala University, Uppsala, Sweden; 4 Department of Clinical Neuroscience, Karolinska Institutet, Stockholm, Sweden; 5 School of Academic Psychiatry, Institute of psychiatry, Psychology & Neuroscience, King’s College London, London, United Kingdom

**Keywords:** executive functions (EF), cognition, psychiatry, transdiagnostic, dimensional, categorical diagnosis, precision medicine

## Introduction: the translational imperative

The primary purpose of psychiatric brain sciences is translational - to generate insights that translate into better treatments and improved lives. Yet for decades our field has been constrained by a disorder-dysfunction paradigm that presents diagnostic categories as underpinned by a distinctive and discrete core set of neuropsychological deficits which can serve as a treatment target (1). For example, depression may be hypothesized to arise from dysregulated mood networks, ADHD from disrupted attention networks, autism from deficits in social processing networks, and so on. The translational process of how basic neuropsychological science is translated to clinical innovation within such a framework is clear: identify the deficit, design the intervention to target that deficit and resolve the disorder caused by the deficit.

## The need to move beyond diagnosis-specific neuropsychological deficits: overlap and heterogeneity

Frankly this model has not delivered. Despite substantial scientific progress more generally in understanding the brain-basis of different conditions, translational progress built on the categorical framework has yielded limited translational progress (2–7). New science-derived treatments, such as brain training and neuromodulation, remain blunt, effect sizes in RCTs are at best small, so that many patients continue to struggle without meaningful relief (8, 9). The promise of disorder-specific deficits as direct targets has proven largely illusory. Progress in brain sciences emerging from our laboratories now points to a very different reality: overlap between conditions and striking heterogeneity within them (10–12).

Across psychiatry, evidence has accumulated to challenge the credibility of this paradigm. Many neuropsychological deficits and associated brain alterations recur across ADHD, autism, mood, anxiety, and personality disorders (13). At the same time, patients given the same diagnostic label often differ from one another profoundly in cognitive profile, trajectory, and treatment response. ADHD provides a striking example. Only a subset of individuals with ADHD show objectively measured deficits in executive functions (EFs) (14, 15); others may present with for example motivational or emotional dysregulation as primary drivers (1, 16).

This inter-individual and cross-condition variation may be regarded as irrelevant noise obscuring the underlying true phenotype. Alternatively, it may not be the noise but the true signal – capturing the true nature of the complexity of how brain processes are mapped onto clinical conditions? An alternative to only rely on categorical diagnoses is to construct models that can capture these causal variations and overlaps. A dimensional approach may be a first step to characterize inter-individual differences (16–20). However, we believe it is not enough to just rebrand categories as dimensions. Rather, psychiatric science needs to pursue greater precision that can drive therapeutic innovation by identifying mechanistic processes that cut across diagnostic boundaries while also parsing variation within them. Through this it can provide more powerful targets for stratification and intervention—bringing us closer to truly personalised psychiatry.

## Executive function as a translational candidate

Among candidate constructs, executive function (EF), both in its generality as an umbrella construct, but also in its specificity offers a compelling focus. EFs—working memory, inhibition, cognitive flexibility, planning—are the top-down control processes that govern behaviour and enable adaptation to complex environments (21, 22). They represent a plausible mechanistic substrate through which heterogeneity and overlap may be understood.

Crucially, EF deficits are not universal within any one condition. In the subset of patients with ADHD that exhibit profound EF impairment, these deficits are strongly linked to poor functional outcomes—educational underachievement, occupational difficulties, interpersonal conflict (14, 15). In depression, anxiety, and bipolar disorder, EF difficulties predict illness severity, degree of dysfunction, relapse risk, and treatment non-response (23–26). In autism, EF profiles help explain variability in adaptive functioning and comorbid psychopathology (27). Across disorders, then, EF impairments mark out subgroups with distinct trajectories and treatment needs.

## Evidence from recent studies

The contributions in this Research Topic further illustrate the translational promise—and challenges—of EF research. Kaiser et al. show that while EF profiles may not predict stimulant response at the individual level, they nonetheless capture mechanistic variation relevant for treatment development. Farnes et al. highlight the complexities of targeting EF with neuromodulation approaches such as rTMS. Sacu et al. and Cully and Björnsdotter underscore how environmental adversity and stress may shape EF-related neural circuits, identifying early prevention targets. Vestberg et al. demonstrate that cognitive flexibility predicts resilience and reduced sick leave, linking EF capacities directly to real-world outcomes. Finally, Malekizadeh et al. show that EF deficits align more closely with depression severity than categorical diagnosis, and may represent a vulnerability as well as a consequence.

Taken together, these studies highlight EF as a *translational bridge*: a cognitive construct that both reflects mechanistic heterogeneity within conditions and spans across traditional diagnostic boundaries.

## Implications for translational psychiatry

Positioning EF at the heart of translational psychiatry carries three major implications:

1. Stratification: EF measures can parse heterogeneity within disorders, identifying clinically relevant subgroups.
2. Prediction: EF performance and neural markers can guide prognosis and treatment selection.
3. Intervention development: By targeting EF directly—through cognitive training, pharmacological modulation, or environmental scaffolding—we can design interventions that cut across categories.

If psychiatric science is to progress, it must move beyond the disorder paradigm that defines its current practice and its search for the illusory “core deficits”. The science emerging demands a new model: one that captures the causal heterogeneity within and overlap across disorders and translates this knowledge into new targets for new treatments. Executive functions provide a uniquely powerful window onto this challenge. By embedding EF research into translational pipelines, psychiatry can begin to deliver on its long-standing promise: personalised, mechanism-based interventions that make a tangible difference in people’s lives.
